# Interval-valued intuitionistic fuzzy multi-attribute group decision-making method considering risk preference of decision-makers and its application

**DOI:** 10.1038/s41598-022-15815-1

**Published:** 2022-07-08

**Authors:** Sha Fu, Ye-zhi Xiao, Hang-jun Zhou

**Affiliations:** School of Information Technology and Management, Hunan University of Finance and Economics, No.139, Section 2, Fenglin Road, Yuelu District, Changsha, 410205 China

**Keywords:** Engineering, Mathematics and computing

## Abstract

An improved interval-valued intuitionistic fuzzy multi-attribute group decision-making method considering the risk preference of decision-makers is proposed to solve the multi-attribute group decision-making problem with interval-valued intuitionistic fuzzy numbers and the condition that the attribute weight information is completely unknown. Firstly, the decision-maker weight of each attribute is determined by combining similarity and proximity. In order to consider the influence of the decision-maker's risk preference on the decision result and avoid the asymptotic behavior of interval-valued intuitionistic fuzzy matrix, the risk aversion coefficient of the decision-maker is introduced and combined with the determined decision-maker's weight aggregation to form a group decision matrix. Then, the information of group decision matrix is mined, and the interval-valued intuitionistic fuzzy entropy is used to determine the attribute weight and relative weight. Based on the interval-valued intuitionistic fuzzy distance measure formula and the TODIM method, the overall superiority of each scheme relative to other schemes is obtained by calculating the superiority between schemes, and the optimal scheme is determined by comparing and sequencing. Finally, the rationality and effectiveness of the proposed method are verified by an example of mechanical assembly supplier selection decision.

## Introduction

Most of the decision-making problems in the fields of sociology, economics, engineering and information science are complex and highly fuzzy. In practice, the classical fuzzy set theory is limited because it expresses less intrinsic information. As a result, Atanassov extended the fuzzy set theory in 1986, and put forward the intuitionistic fuzzy sets (IFS) theory^1^, that is, by introducing concepts such as non-membership degree and hesitation degree, the fuzziness of things can be analyzed more deeply and carefully, which is the most influential extension and development of the fuzzy set theory. To solve the problem that information such as membership degree and non-membership degree cannot be characterized by quasi-exact values sometimes, Atanassov and Gargov^2^ further extended the IFS, proposed the concept of interval-valued intuitionistic fuzzy sets (IVIFS), and defined the basic algorithm of IVIFS. Because IVIFS have the advantage that both membership and non-membership are interval values, which can describe fuzziness more flexibly, relevant theories are used to solve multi-attribute decision-making (MADM) problems such as investment evaluation, logic planning, pattern recognition and machine learning^3–5^.

At present, some scholars have carried out research around the four key links of interval-valued intuitionistic fuzzy decision making, including attribute weight determination, decision-maker weight determination, similarity measure theory and decision-making method, and have achieved some results. In determining attribute weights, Yang et al*.*^6^ used interval-valued intuitionistic fuzzy uncertain language variables to model uncertain information, and used maximum deviation method to establish a linear programming model to calculate attribute weight vectors. Xu et al*.*^7^ used fuzzy clustering method to cluster the expert preference of each stage and determined the weight of each attribute based on fuzzy entropy. With incomplete information on attribute weights, Wan and Dong^8^ proposed a bi-objective linear programming model that satisfies both non-consistency minimization and consistency maximization to determine attribute weights. Yu et al.^9^ developed a new and unified intuitionistic fuzzy multi-objective linear programming model for such portfolio selection problems. In case of completely unknown attribute weights, Jin et al.^10^ defined the information entropy based on interval-valued intuitionistic fuzzy number (IVIFN) to determine the attribute weights. Xu and Shen^11^ proposed an entropy measure for IVIFS and establish an entropy weight model, which is then used to determine the objective attribute weights of the alternatives. Regarding the determination of decision-maker weights, Zhang and Xu^12^ proposed a goal programming method based on incomplete decision-maker weight information to determine decision-maker weights. Wan and Dong^13^ proposed a method to determine the weights of decision-makers based on similarity. The above research methods have no difference on different attributes of decision-makers' weights, which is not in line with the reality, because in practical decision-making problems, every decision-maker is often only good at certain fields but not all fields. Therefore, it is more realistic and reasonable to assign the decision-maker weight for each attribute to each decision-maker. In summary, there is a lack of detailed and in-depth discussion on how to set the weight of decision-makers objectively and reasonably.

In the research on the similarity measure of IFS, Khalid and Abbas^14^ defined a similarity measure of intuitionistic fuzzy soft sets based on Hausdorff distance, and extended this application to medical diagnosis. Zhang and Yu^15^ proposed the similarity measure and the distance measure of IVIFS, which considered all the information on IVIFS and effectively overcome the information loss. Joshi and Kumar^16^ studied the decision-making problem of IVIFS by calculating the similarity between each scheme and ideal scheme and extending Hamming distance. In the optimization of quantization methods for decision analysis, Behret^17^ gave the conditions for additive consistency and multiplicative consistency of intuitionistic fuzzy preference relations by using the relationship between interval fuzzy sets and IFS, and established an optimization model to obtain the priority of decision schemes. Pang and Song^18^ proposed a hybrid weighted aggregation method by fusing the objective comprehensive weights of experts and the weights of individual comprehensive evaluation values based on similarity, so as to obtain the group comprehensive evaluation values of schemes, and realized the ranking of schemes by defining the expected values with risk attitude factors and accurate functions. To derive priority weights of alternatives, Wan et al*.*^19^ established an Atanassov intuitionistic fuzzy programming model, and solved by three approaches considering decision makers’ different risk attitudes. Zhang^20^ described the multi-stage bilateral matching problem under the intuitionistic fuzzy preference information, and constructed a stage matching weight optimization model based on the intuitionistic fuzzy similarity. Zhang et al*.*^21^ constructed the evaluation criterion system of equipment supplier selection based on military supply chain, and proposed a group decision method based on intuitionistic fuzzy entropy and extended multi-criteria compromise solution framework.

To sum up, the above methods are very effective in solving multi-attribute group decision-making (MAGDM) problems based on IVIFN, but they also have some limitations, because different decision-makers often have different risk preferences in actual decision-making. Shi and Zhang^22^ proposed an improved method of additive consistency of intuitionistic fuzzy preference relations based on decision error transfer formula for decision-making problems in the context of intuitionistic fuzzy preference information. Zhang et al*.*^23^ based on the objective situation that different experts have different risk attitudes towards the same decision-making problem, introduced a risk preference coefficient to describe the different preferences of decision-makers for the uncertainty information, and constructed a ranking method from the perspectives of decision-makers' risk attitudes and similarity measures of IFS. Wang and Wan^24^ investigated the group decision making with interval-valued intuitionistic fuzzy preference relations, and proposed a new order relation to rank interval-valued intuitionistic fuzzy values. Garcez et al.^25^ put forward a new hybrid Grey Additive-Veto Model (GAVM) for selecting suppliers, which optimizes the choice by the decision-maker's preference. Decision-makers with different risk preferences for the same MADM problem may make different decisions, so it is necessary to consider the risk preferences of decision-makers in the process of MADM. However, the multi-attribute scheme ranking method proposed in the reference^11^ does not consider the impact of the risk preferences of decision-makers on the decision results. In addition, although the fuzziness of decision-making environment is considered in the above references^17,20,21^, the psychological behavior of decision-makers is seldom considered, and some studies, although involving the psychological behavior of decision-makers, do not consider the risk attitude of decision-makers, which affects the validity and accuracy of decision-making results. In view of the fact that TOmada de decisão interativa multicritério (TODIM) method^26^ is a decision-making method which is close to the preference of decision-makers on the basis of considering the psychological behavior of decision-makers, the combination of TODIM method in this study can better reflect the subjective risk preference of decision-makers, without giving the information of decision reference points in advance, and can retain the decision-making information more completely.

Global economic integration and the development of information technology also make enterprises face more intense market competition. Supply chain management, as a new management mode adapted to global manufacturing and diversified customer needs, has been widely used in enterprises. If one or several companies in the supply chain experience production blockages, changes in delivery dates, and increases in costs, the responsiveness of the entire supply chain will deteriorate, and the total cost of the supply chain will also increase.

The issue of supplier selection is an important part of supply chain management and a prerequisite for doing a good job in supply chain management^27^, which has always attracted the attention of theoretical and practical management workers, because the selection of excellent suppliers is of great significance for enterprises to reduce costs, reduce risks and enhance market competitiveness. According to the instance analysis given in this paper, the decision-making problem of mechanical assembly suppliers is essentially the research of MAGDM under uncertain conditions. Therefore, in this study, considering the fuzziness, complexity and urgency of the above-mentioned decision-making problems, a decision-making model based on IVIFS and TODIM method is established by combining the fuzziness of decision-making environment with the psychological behavior and risk attitude of decision-makers, and the robustness of the model is verified by different interval-valued intuitionistic fuzzy distance measurement formulas, so as to improve the scientificity and rationality of MAGDM under the risk preference state of decision-makers. Therefore, the supplier selection problem is studied as a group decision-making problem in interval-valued intuitionistic fuzzy environment, which is closer to the actual decision-making background, the research results of this paper have certain theoretical value and practical application value.

## Preliminaries

### Interval-valued intuitionistic fuzzy numbers and operational rule

#### Definition 1

If $X$ is a non-empty set, then

$$A = \{ < x,\mu_{A} (x),\nu_{A} (x) > |x \in X\}$$

is an intuitionistic fuzzy set, in which $\mu_{A} (x)$ and $\nu_{A} (x)$ are respectively membership and non-membership of element $x$ in $X$ belonging to $A$, $\mu_{A} (x) \in [0,1]$, $\nu_{A} (x) \in [0,1][0,1]$, and meeting $0 \le \mu_{A} (x) + \nu_{A} (x) \le 1$, $x \in X$.

Besides, $\pi_{A} (x) = 1 - \mu_{A} (x) - \nu_{A} (x)$, indicating that the hesitancy degree or uncertainty of element $x$ in $X$ belonging to $A$^28^.

#### Definition 2

If $X$ is a non-empty set, then

$$\tilde{A} = \{ < x,\tilde{\mu }_{{\tilde{A}}} (x),\tilde{\nu }_{{\tilde{A}}} (x) > |x \in X\}$$

is an intuitionistic fuzzy set, in which $\tilde{\mu }_{{\tilde{A}}} (x) \subset [0,1]$, $\tilde{\nu }_{{\tilde{A}}} (x) \subset [0,1]$,$x \in X$ and

$$\sup \tilde{\mu }_{{\tilde{A}}} (x) + \sup \tilde{\nu }_{{\tilde{A}}} (x) \le 1,\,x \in X.$$

Interval numbers $\tilde{\mu }_{{\tilde{A}}} (x)$ and $\tilde{\nu }_{{\tilde{A}}} (x)$ are respectively membership and non-membership of element $x$ in $X$ belonging to $\tilde{A}$.

$$\tilde{\mu }_{{\tilde{A}}} (x) = [\tilde{\mu }_{{\tilde{A}}}^{L} (x),\tilde{\mu }_{{\tilde{A}}}^{U} (x)],\,\tilde{\nu }_{{\tilde{A}}} (x) = [\tilde{\nu }_{{\tilde{A}}}^{L} (x),\tilde{\nu }_{{\tilde{A}}}^{U} (x)].$$

Then IVIFS $\tilde{A}$ can be recorded as:

$$\tilde{A} = \{ < x,[\tilde{\mu }_{{\tilde{A}}}^{L} (x),\tilde{\mu }_{{\tilde{A}}}^{U} (x)],[\tilde{\nu }_{{\tilde{A}}}^{L} (x),\tilde{\nu }_{{\tilde{A}}}^{U} (x)] > |x \in X\} .$$

Thus $\tilde{\pi }_{{\tilde{A}}} (x) = [\tilde{\pi }_{{\tilde{A}}}^{L} (x),\tilde{\pi }_{{\tilde{A}}}^{U} (x)]$ is the hesitancy degree of element $x$ belonging to $\tilde{A}$^29^.

Where

$$\tilde{\pi }_{{\tilde{A}}}^{L} (x) = 1 - \tilde{\mu }_{{\tilde{A}}}^{U} (x) - \tilde{\nu }_{{\tilde{A}}}^{U} (x),\,\tilde{\pi }_{{\tilde{A}}}^{U} (x) = 1 - \tilde{\mu }_{{\tilde{A}}}^{L} (x) - \tilde{\nu }_{{\tilde{A}}}^{L} (x)$$

is a simplified form, and $\tilde{\alpha } = ([a,b], \; [c,d])$ is an IVIFN, where, $[a,b] \subset [0,1]$, $[c,d] \subset [0,1]$, $b + d \le 1$.

#### Definition 3

If $\tilde{\alpha } = ([a,b],[c,d])$ is an IVIFN on the IVIFS $\tilde{A}$, then

1 $$S(\tilde{\alpha }) = \frac{1}{2}(a - c + b - d)$$

is the score of $\tilde{\alpha }$, where $S$ is the score function of $\tilde{\alpha }$, $S(\tilde{\alpha }) \in [ - 1,1]$. Obviously, the greater $S(\tilde{\alpha })$, the greater $\tilde{\alpha }$^30^. Specifically, if $S(\tilde{\alpha }) = 1$, then $\tilde{\alpha }$ takes the maximum $([1,1],[0,0])$; if $S(\tilde{\alpha }) = - 1$, $\tilde{\alpha }$ takes the minimum $([0,0],[1,1])$.

#### Definition 4

If $X$ is a non-empty set, $\tilde{A}_{1} \in \tilde{\Phi }(X)$ and $\tilde{A}_{2} \in \tilde{\Phi }(X)$ are IVIFS. If $d$ is a map: $d:(\tilde{\Phi }(X))^{2} \to [0,1]$, then the distance measure $d(\tilde{A}_{1} ,\tilde{A}_{2} )$ between IVIFS $\tilde{A}_{1}$ and $\tilde{A}_{2}$. Where, $d(\tilde{A}_{1} ,\tilde{A}_{2} )$ meets the following properties^28^:

1. $$0 \le d(\tilde{A}_{1} ,\tilde{A}_{2} ) \le 1$$;
2. $d(\tilde{A}_{1} ,\tilde{A}_{2} ) = 0$, if and only if $\tilde{A}_{1} = \tilde{A}_{2}$;
3. $$d(\tilde{A}_{1} ,\tilde{A}_{2} ) = d(\tilde{A}_{2} ,\tilde{A}_{1} )$$.

To effectively measure the difference degree of IVIFN, Xu^31^ summarized the common interval-valued intuitionistic fuzzy distance measures, and extended the standard Hamming distance and the standard Euclidean distance based on Hausdorff measure.

1. Standard Hamming distance based on Hausdorff measure, hereinafter referred to as $D_{HH}$
  2 $$D_{HH} (\tilde{A}_{i} ,\tilde{A}_{k} ) = \frac{1}{n}\sum\limits_{j = 1}^{n} {\max \{ |\tilde{\mu }_{{\tilde{A}_{i} }}^{L} (x_{j} ) - \tilde{\mu }_{{\tilde{A}_{k} }}^{L} (x_{j} )|,|\tilde{\mu }_{{\tilde{A}_{i} }}^{U} (x_{j} ) - \tilde{\mu }_{{\tilde{A}_{k} }}^{U} (x_{j} )|,|\tilde{\nu }_{{\tilde{A}_{i} }}^{L} (x_{j} ) - \tilde{\nu }_{{\tilde{A}_{k} }}^{L} (x_{j} )|,|\tilde{\nu }_{{\tilde{A}_{i} }}^{U} (x_{j} ) - \tilde{\nu }_{{\tilde{A}_{k} }}^{U} (x_{j} )|\} } .$$
2. Standard Euclidean distance based on Hausdorff measure, hereinafter referred to as $D_{HE}$
  3 $$D_{HE} (\tilde{A}_{i} ,\tilde{A}_{k} ) = \left[ {\frac{1}{n}\sum\limits_{j = 1}^{n} {\max \{ (\tilde{\mu }_{{\tilde{A}_{i} }}^{L} (x_{j} ) - \tilde{\mu }_{{\tilde{A}_{k} }}^{L} (x_{j} ))^{2} ,(\tilde{\mu }_{{\tilde{A}_{i} }}^{U} (x_{j} ) - \tilde{\mu }_{{\tilde{A}_{k} }}^{U} (x_{j} ))^{2} ,(\tilde{\nu }_{{\tilde{A}_{i} }}^{L} (x_{j} ) - \tilde{\nu }_{{\tilde{A}_{k} }}^{L} (x_{j} ))^{2} ,(\tilde{\nu }_{{\tilde{A}_{i} }}^{U} (x_{j} ) - \tilde{\nu }_{{\tilde{A}_{k} }}^{U} (x_{j} ))^{2} \} } } \right]^{\frac{1}{2}} .$$

#### Definition 5

If $\tilde{\alpha } = ([a_{1} ,b_{1} ],[c_{1} ,d_{1} ])$ and $\tilde{\beta } = ([a_{2} ,b_{2} ],[c_{2} ,d_{2} ])$ are two random IVIFN, their operational rules are^32^:

1. $$\tilde{\alpha } \oplus \tilde{\beta } = ([a_{1} + a_{2} - a_{1} a_{2} ,b_{1} + b_{2} - b_{1} b_{2} ],[c_{1} c_{2} ,d_{1} d_{2} ])$$;
2. $$\tilde{\alpha } \otimes \tilde{\beta } = ([a_{1} a_{2} ,b_{1} b_{2} ],[c_{1} + c_{2} - c_{1} c_{2} ,d_{1} + d_{2} - d_{1} d_{2} ])$$;
3. $$\lambda \tilde{\alpha } = ([1 - (1 - a_{1} )^{\lambda } ,1 - (1 - b_{1} )^{\lambda } ],[c_{1}^{\lambda } ,d_{1}^{\lambda } ])$$, $$\lambda > 0$$

#### Definition 6

If $\tilde{\alpha } = ([a_{1} ,b_{1} ],[c_{1} ,d_{1} ])$ and $\tilde{\beta } = ([a_{2} ,b_{2} ],[c_{2} ,d_{2} ])$ are two random IVIFN, the distance between them is expressed as^33^:

4 $$d(\tilde{\alpha },\tilde{\beta }) = \sqrt {\frac{1}{4}[(a_{1} - a_{2} )^{2} + (b_{1} - b_{2} )^{2} + (c_{1} - c_{2} )^{2} + (d_{1} - d_{2} )^{2} + (\pi_{{\tilde{\alpha }}}^{L} - \pi_{{\tilde{\beta }}}^{L} )^{2} + (\pi_{{\tilde{\alpha }}}^{U} - \pi_{{\tilde{\beta }}}^{U} )^{2} ]} ,$$

where, $$\pi_{{\tilde{\alpha }}}^{L} = 1 - b_{j} - d_{j}$$, $$\pi_{{\tilde{\alpha }}}^{U} = 1 - a_{j} - c_{j}$$ $$(j = 1)$$.

$$\pi_{{\tilde{\beta }}}^{L} = 1 - b_{j} - d_{j} ,\,\pi_{{\tilde{\beta }}}^{U} = 1 - a_{j} - c_{j} (j = 2).$$

### Interval-valued intuitionistic fuzzy integration operator and asymptotic property

#### Definition 7

If $\tilde{\alpha }_{j} = ([a_{j} ,b_{j} ],[c_{j} ,d_{j} ])$ $(j = 1,2, \ldots ,n)$ is an IVIFN^34^, and $IVIFWM:Q^{n} \to Q$, if

5 $$\begin{aligned} IVIFWM_{w} (\tilde{\alpha }_{1} ,\tilde{\alpha }_{2} , \ldots ,\tilde{\alpha }_{n} ) & = \sum\limits_{j = 1}^{n} {w_{j} \tilde{\alpha }_{j} } \\ & = ([1 - \prod\limits_{j = 1}^{n} {(1 - a_{j} )^{{w_{j} }} } ,1 - \prod\limits_{j = 1}^{n} {(1 - b_{j} )^{{w_{j} }} } ],[\prod\limits_{j = 1}^{n} {c_{j}^{{w_{j} }} } ,\prod\limits_{j = 1}^{n} {d_{j}^{{w_{j} }} } ]), \\ \end{aligned}$$

where, $Q$ is the set of all IVIFN, $w = (w_{1} ,w_{2} , \ldots ,w_{n} )^{T}$ is the weight vector of $\tilde{\alpha }_{j}$ $(j = 1,2, \ldots ,n)$, meeting $w_{j} \in [0,1]$ and $\sum\nolimits_{j = 1}^{n} {w_{j} = 1}$, then interval-valued intuitionistic fuzzy weighted mean (IVIFWM) is the weighted arithmetic mean operator of IVIFN^35^.

## Determination of decision-makers' weights and solution of attribute weights

### Determination of decision-makers' weights for attributes

Due to the differences in background knowledge and information symmetry among decision-makers, as well as the complexity and uncertainty of decision-making problems, preference conflicts inevitably exist among decision-makers. Because different decision-makers have different knowledge structures, personal preferences, and different understanding of schemes and attributes, the evaluation results will also be very different, so how to determine the appropriate weights of decision-makers has become the key to decision-making.

For each attribute $C_{j}$, the evaluation values of all schemes given by the decision-maker $E_{k}$ about the attribute are expressed as interval-valued intuitionistic fuzzy vector $\tilde{r}_{j}^{k} = (\tilde{r}_{1j}^{k} ,\tilde{r}_{2j}^{k} , \ldots ,\tilde{r}_{mj}^{k} )$, where $\omega_{j}^{k}$ represents the decision-maker's weight about the attribute $C_{j}$ of decision-maker $E_{k}$. In order to determine the value of the decision-maker's weight, two aspects need to be considered at the same time, one is similarity, which is used to indicate the similarity between the individual decision matrix of decision-maker $E_{k}$ and the group decision matrix composed of all decision-makers, and the other is proximity, which is used to indicate the proximity between the individual decision matrix of decision-maker $E_{k}$ and the group decision matrix composed of all other decision-makers except the decision-maker $E_{k}$^36^. According to the above analysis, the main steps to solve the decision-maker weight $\omega_{j}^{k}$ of each attribute are as follows:

1. Determine the positive ideal solution vector $\tilde{r}_{j}$ under attribute $C_{j}$, namely $\tilde{r}_{j} = (\tilde{r}_{1j} ,\tilde{r}_{2j} , \ldots ,\tilde{r}_{mj} )$.

As we all know, most group decisions usually take the average evaluation values of multiple decision makers as the final group decision result. Therefore, the closer the evaluation value given by a decision maker is to the average value, the better the evaluation value is. On the contrary, the farther away it is from the average value, the worse the evaluation value is. Hence, this study selects the interval-valued intuitionistic fuzzy mean (IVIFM) provided by all decision makers under the attribute $$C_{j}$$ as the positive ideal solution vector $$\tilde{r}_{j}$$. For the attribute $$C_{j}$$ , the evaluation value of all schemes given by the decision maker $$E_{k}$$ with regard to the attribute is expressed as the interval-valued intuitionistic fuzzy vector $$\tilde{r}_{j}^{k} = (\tilde{r}_{1j}^{k} ,\tilde{r}_{2j}^{k} , \ldots ,\tilde{r}_{mj}^{k} )$$, and the positive ideal solution vector $$\tilde{r}_{j}$$ of the attribute $$C_{j}$$ is the arithmetic mean of $$\tilde{r}_{j}^{k}$$$$(k \in K)$$^37^.

6 $$\tilde{r}_{ij} = ([a_{ij}^{*} ,b_{ij}^{*} ],[c_{ij}^{*} ,d_{ij}^{*} ]) = IVIFM_{w} (\tilde{r}_{ij}^{1} ,\tilde{r}_{ij}^{2} , \ldots ,\tilde{r}_{ij}^{s} )$$

where, $$i = 1,2, \ldots ,m$$.

2. Determine all negative ideal solution vectors under attribute $C_{j}$.

The negative ideal solution vectors about the attribute $C_{j}$ include an individual negative ideal solution vector $\tilde{r}_{j}^{c}$, an individual left negative ideal solution vector $\tilde{r}_{j}^{L - }$ and an individual right negative ideal solution vector $\tilde{r}_{j}^{U - }$, which are expressed as: $\tilde{r}_{j}^{c} = (\tilde{r}_{1j}^{c} ,\tilde{r}_{2j}^{c} , \ldots ,\tilde{r}_{mj}^{c} )$,$\tilde{r}_{j}^{L - } = (\tilde{r}_{1j}^{L - } ,\tilde{r}_{2j}^{L - } , \ldots ,\tilde{r}_{mj}^{L - } )$ and $\tilde{r}_{j}^{U - } = (\tilde{r}_{1j}^{U - } ,\tilde{r}_{2j}^{U - } , \ldots ,\tilde{r}_{mj}^{U - } )$.

Where

7 $$\tilde{r}_{ij}^{c} = ([a_{ij}^{c} ,b_{ij}^{c} ],[c_{ij}^{c} ,d_{ij}^{c} ]),\,a_{ij}^{c} = c_{ij}^{*} ,\,b_{ij}^{c} = d_{ij}^{*} ,\,c_{ij}^{c} = a_{ij}^{*} ,\,d_{ij}^{c} = b_{ij}^{*}$$

8 $$\tilde{r}_{ij}^{L - } = ([a_{ij}^{L - } ,b_{ij}^{L - } ],[c_{ij}^{L - } ,d_{ij}^{L - } ]),a_{ij}^{L - } = \mathop {\min }\limits_{k} \{ a_{ij}^{k} \} ,b_{ij}^{L - } = \mathop {\min }\limits_{k} \{ b_{ij}^{k} \} ,\,c_{ij}^{L - } = \mathop {\max }\limits_{k} \{ c_{ij}^{k} \} ,\,a_{ij}^{L - } = \mathop {\min }\limits_{k} \{ a_{ij}^{k} \}$$

9 $$\tilde{r}_{ij}^{U - } = ([a_{ij}^{U - } ,b_{ij}^{U - } ],[c_{ij}^{U - } ,d_{ij}^{U - } ]),a_{ij}^{U - } = \mathop {\max }\limits_{k} \{ a_{ij}^{k} \} ,\,b_{ij}^{U - } = \mathop {\max }\limits_{k} \{ b_{ij}^{k} \} ,\,c_{ij}^{U - } = \mathop {\min }\limits_{k} \{ c_{ij}^{k} \} ,\,d_{ij}^{U - } = \mathop {\min }\limits_{k} \{ d_{ij}^{k} \}$$

3. Calculate the distances $d(\tilde{r}_{j}^{k} ,\tilde{r}_{j} )$, $d(\tilde{r}_{j}^{k} ,\tilde{r}_{j}^{c} )$, $d(\tilde{r}_{j}^{k} ,\tilde{r}_{j}^{L - } )$ and $d(\tilde{r}_{j}^{k} ,\tilde{r}_{j}^{U - } )$ by using formula (4).
4. Calculate the similarity

The similarity between individual decision matrix of decision-maker $E_{k}$ and group decision matrix under attribute $C_{j}$ is expressed by $s_{j}^{k}$^38^:

10 $$s_{j}^{k} = \frac{{d(\tilde{r}_{j}^{k} ,\tilde{r}_{j}^{c} ) + d(\tilde{r}_{j}^{k} ,\tilde{r}_{j}^{L - } ) + d(\tilde{r}_{j}^{k} ,\tilde{r}_{j}^{U - } )}}{{d(\tilde{r}_{j}^{k} ,\tilde{r}_{j} ) + d(\tilde{r}_{j}^{k} ,\tilde{r}_{j}^{c} ) + d(\tilde{r}_{j}^{k} ,\tilde{r}_{j}^{L - } ) + d(\tilde{r}_{j}^{k} ,\tilde{r}_{j}^{U - } )}}$$

where, $$j = 1,2, \ldots ,n$$; $$k = 1,2, \ldots ,s$$.

5. Calculate the proximity $\xi_{ij}^{lk}$ and average proximity $\eta (\tilde{r}_{j}^{l} ,\tilde{r}_{j}^{k} )$ between $\tilde{r}_{ij}^{l}$ and $\tilde{r}_{ij}^{k}$.
  11 $$\xi_{ij}^{lk} = 1 - d(\tilde{r}_{ij}^{l} ,\tilde{r}_{ij}^{k} )$$
  12 $$\eta (\tilde{r}_{j}^{l} ,\tilde{r}_{j}^{k} ) = \frac{1}{m}\sum\limits_{i = 1}^{m} {\xi_{ij}^{lk} }$$

6. For attribute $C_{j}$, the proximity $\eta_{j}^{k}$ between the decision-maker $E_{k}$ and all other decision-makers is:
  13 $$\eta_{j}^{k} = \frac{1}{s - 1}\sum\limits_{l = 1,l \ne k}^{s} {\eta (\tilde{r}_{j}^{l} ,\tilde{r}_{j}^{k} )}$$

7. Calculate the weight of decision-makers for each attribute. Combining the similarity and proximity obtained above, the combination weight $\tilde{\omega }_{j}^{k}$ of decision-maker $E_{k}$ under attribute $C_{j}$ is constructed.
  14 $$\tilde{\omega }_{j}^{k} = \lambda s_{j}^{k} + (1 - \lambda )\eta_{j}^{k}$$

$\lambda$ is the control coefficient and can be weighed by changing the control coefficient between the similarity and proximity. In practical application, $\lambda = 0.5$ can be taken as a compromise value.

For the combination weight $\tilde{\omega }_{j}^{k}$, the weight $\omega_{j}^{k}$ of the decision-maker $E_{k}$ under the attribute $C_{j}$ can be obtained after standardization:

15 $$\omega_{j}^{k} = \frac{{\tilde{\omega }_{j}^{k} }}{{\sum\nolimits_{l = 1}^{s} {\tilde{\omega }_{j}^{l} } }}.$$

### Solution of unknown attribute weights

In the process of MAGDM, attribute weight information is often completely unknown or incomplete, due to time constraints, lack of relevant knowledge and limited knowledge of the increasingly complex and uncertain group decision-making environment. In view of the fact that the attribute weight information is completely unknown in this study, the group decision matrix information is deeply mined, and the weight $w_{j}$ and relative weight $w^{\prime}_{j}$ of each attribute are calculated by using interval-valued intuitionistic fuzzy entropy.

Interval-valued intuitionistic fuzzy entropy is defined as:

16 $$E(C_{j} ) = \frac{1}{m}\sum\limits_{i = 1}^{m} {\frac{{\min (\tilde{\mu }_{{\tilde{A}}}^{L} (x),\tilde{\nu }_{{\tilde{A}}}^{L} (x)) + \min (\tilde{\mu }_{{\tilde{A}}}^{U} (x),\tilde{\nu }_{{\tilde{A}}}^{U} (x)) + \tilde{\pi }_{{\tilde{A}}}^{L} (x) + \tilde{\pi }_{{\tilde{A}}}^{U} (x)}}{{\max (\tilde{\mu }_{{\tilde{A}}}^{L} (x),\tilde{\nu }_{{\tilde{A}}}^{L} (x)) + \max (\tilde{\mu }_{{\tilde{A}}}^{U} (x),\tilde{\nu }_{{\tilde{A}}}^{U} (x)) + \tilde{\pi }_{{\tilde{A}}}^{L} (x) + \tilde{\pi }_{{\tilde{A}}}^{U} (x)}}} ,$$

where, $$\tilde{\pi }_{{\tilde{A}}}^{L} (x) = 1 - \tilde{\mu }_{{\tilde{A}}}^{U} (x) - \tilde{\nu }_{{\tilde{A}}}^{U} (x)$$, $$\tilde{\pi }_{{\tilde{A}}}^{U} (x) = 1 - \tilde{\mu }_{{\tilde{A}}}^{L} (x) - \tilde{\nu }_{{\tilde{A}}}^{L} (x)$$.

17 $$w_{j} = \frac{{1 - E(C_{j} )}}{{\sum\nolimits_{j = 1}^{n} {(1 - E(C_{j} ))} }}.$$

The relative weight of attribute $w^{\prime}_{j}$ is:

18 $$w^{\prime}_{j} = \frac{{w_{j} }}{{w_{\max } }}.$$

When the attribute information data of each scheme is an IVIFN, from the perspective of reflecting the original decision information, the more fuzzy or uncertain the attribute information is, the less information the attribute has available for the scheme is, and the larger the entropy value is, the smaller the weight should be given, and vice versa^39^. Therefore, interval-valued intuitionistic fuzzy entropy can be used to determine the weight of attributes to not only reduce the loss of evaluation information but also truly reflect the wishes of decision-makers.

## Interval-valued intuitionistic fuzzy multi-attribute group decision-making method considering risk preference of decision-makers

### Description of supplier selection decision-making problem

In the MAGDM problem of supplier selection, a joint evaluation team composed of production, procurement, logistics and other departments of the enterprise assigns values to the subjective and objective attributes of the selected mixed evaluation information, and ranks and selects the best suppliers according to the qualitative evaluation information given by each decision-making department and the quantitative evaluation information obtained through statistical analysis. For the MAGDM problem in this study, $s$ experts have evaluated $n$ attributes of $m$ schemes. Let the scheme set be $A = \{ A_{1} ,A_{2} , \ldots ,A_{m} \}$, where $A_{i}$ is the $i$th scheme, $i = 1,2, \ldots ,m$; evaluation attribute set is $C = \{ C_{1} ,C_{2} , \ldots ,C_{n} \}$, where $C_{j}$ is the $j$th attribute of the scheme, $j = 1,2, \ldots ,n$. $E = \{ E_{1} ,E_{2} , \ldots ,E_{s} \}$ is the set of decision-making experts, and $W = \{ w_{1} ,w_{2} , \ldots ,w_{n} \}$ indicates the attribute weight vector that meets $0 \le w_{j} \le 1$ and $\sum\nolimits_{j = 1}^{n} {w_{j} } = 1$. $[a_{ij}^{k} ,b_{ij}^{k} ]$ and $[c_{ij}^{k} ,d_{ij}^{k} ]$ respectively indicate the membership interval (satisfaction degree) or non-membership interval (dissatisfaction degree) of the decision-maker $E_{k}$ on the scheme $A_{i}$ about attribute $C_{j}$, $k = 1,2, \ldots ,s$, where, $[a_{ij}^{k} ,b_{ij}^{k} ] \subseteq [0,1]$,$[c_{ij}^{k} ,d_{ij}^{k} ] \subseteq [0,1]$, $b_{ij}^{k} + d_{ij}^{k} \le 1$. The evaluation value of the decision-maker $E_{k}$ on the attribute $C_{j}$ of the scheme $A_{i}$ is the IVIFN, which is expressed as $\tilde{r}_{ij}^{k} = ([a_{ij}^{k} ,b_{ij}^{k} ],[c_{ij}^{k} ,d_{ij}^{k} ])$. The interval-valued intuitionistic fuzzy decision matrix composed of the above evaluation values is written as $\tilde{R}^{k} = (\tilde{r}_{ij}^{k} )_{m \times n}$.

### Algorithm flow

Based on the above analysis, the flow of MAGDM method based on IVIFN is as follows:

**Step 1:** All decision-makers $E_{k}$ give their corresponding interval-valued intuitionistic fuzzy matrices $\tilde{R}^{k} = (\tilde{r}_{ij}^{k} )_{m \times n}$.

**Step 2:** According to formulas (14)–(15), the decision-maker weight $\omega_{j}^{k}$ of the decision-maker $E_{k}$ about each attribute $C_{j}$ is calculated.

**Step 3:** The n-dimensional IVIFWM operator^40^ is used in combination with the decision-maker weight determined in Step 2 to aggregate the single decision matrix $\tilde{R}^{k} = (\tilde{r}_{ij}^{k} )_{m \times n}$ of the decision-maker and get the group decision matrix $\tilde{R} = (\tilde{r}_{ij} )_{m \times n}$.

19 $$\tilde{r}_{ij} = ([a_{ij} ,b_{ij} ],[c_{ij} ,d_{ij} ]) = \sum\limits_{k = 1}^{s} {\omega_{j}^{k} \tilde{r}_{ij}^{k} } = ([\sum\limits_{k = 1}^{s} {\omega_{j}^{k} a_{ij}^{k} } ,\sum\limits_{k = 1}^{s} {\omega_{j}^{k} b_{ij}^{k} } ],[\sum\limits_{k = 1}^{s} {\omega_{j}^{k} c_{ij}^{k} } ,\sum\limits_{k = 1}^{s} {\omega_{j}^{k} d_{ij}^{k} } ]).$$

It is obvious that the aggregated group decision matrix is still an interval-valued intuitionistic fuzzy matrix.

**Step 4:** According to formulas (17)–(18), the weight $w_{j}$ and relative weight $w^{\prime}_{j}$ of each attribute are calculated by using interval-valued intuitionistic fuzzy entropy.

**Step 5:** The dominance $\varphi_{j} (A_{i} ,A_{k} )$ of scheme $A_{i}$ relative to scheme $A_{k}$ under attribute $C_{j}$ is calculated.

20 $$\varphi_{j} (A_{i} ,A_{k} ) = \left\{ {\begin{array}{*{20}c} {\sqrt {\frac{{w^{\prime}_{j} }}{{\sum\limits_{j = 1}^{n} {w^{\prime}_{j} } }}D(r_{ij} ,r_{kj} )} } & {S_{ij} (\tilde{\alpha }) > S_{kj} (\tilde{\alpha })} \\ 0 & {S_{ij} (\tilde{\alpha }) = S_{kj} (\tilde{\alpha })} \\ { - \frac{1}{\theta }\sqrt {\frac{{\sum\limits_{j = 1}^{n} {w^{\prime}_{j} } }}{{w^{\prime}_{j} }}D(r_{ij} ,r_{kj} )} } & {S_{ij} (\tilde{\alpha }) < S_{kj} (\tilde{\alpha }).} \\ \end{array} } \right.$$

Here, the dominance matrix $[\varphi_{j} (A_{i} ,A_{k} )]_{m \times m}$ under attribute $C_{j}$ condition is constructed. In formula (20), $S_{ij} (\tilde{\alpha })$ and $S_{kj} (\tilde{\alpha })$ are the score functions of schemes $A_{i}$ and $A_{k}$ under attribute $C_{j}$, respectively, and parameter $\theta$ is the risk aversion coefficient, which can be changed according to the preference of decision-makers, ranging $0 \le \theta \le (\sum\nolimits_{j = 1}^{n} {w_{j}^{\prime } } )/w_{j}^{\prime }$, and the smaller the value of $\theta$, the higher the degree of risk aversion of decision-makers^41^.

**Step 6:** According to the dominance matrix, the overall dominance of scheme $A_{i}$ relative to scheme $A_{k}$ is calculated.

21 $$\varphi (A_{i} ,A_{k} ) = \sum\limits_{j = 1}^{n} {\varphi_{j} (A_{i} ,A_{k} )} ,$$

where, $$i,k = 1,2, \ldots ,m$$.

**Step 7:** The overall dominance $P(A_{i} )$ of scheme $A_{i}$ relative to other schemes is comprehensively calculated, and the schemes are sequenced according to their values to get the comprehensive evaluation values of each scheme and to select the optimal scheme.

22 $$P(A_{i} ) = \frac{{\sum\nolimits_{k = 1}^{m} {\varphi (A_{i} ,A_{k} )} - \min_{1 \le i \le m} \{ \sum\nolimits_{k = 1}^{m} {\varphi (A_{i} ,A_{k} )} \} }}{{\max_{1 \le i \le m} \{ \sum\nolimits_{k = 1}^{m} {\varphi (A_{i} ,A_{k} )} \} - \min_{1 \le i \le m} \{ \sum\nolimits_{k = 1}^{m} {\varphi (A_{i} ,A_{k} )} \} }}$$

## Instance analysis

A regional manufacturing company is looking for the best supplier in the world for the most critical components in its assembly process. There are four suppliers $A_{i} (i = 1,2,3,4)$ to choose from. An expert group composed of four experts (decision-makers) $E_{k} (k = 1,2,3,4)$ from various strategic decision-making fields evaluates suppliers $A_{i}$ with the following five evaluation indicators (attributes): product price $(C_{1} )$, product quality $(C_{2} )$, supplier maintenance level $(C_{3} )$, supplier information $(C_{4} )$ and risk factors $(C_{5} )$. Decision-makers $E_{k}$ use the IVIFN $\tilde{r}_{ij}^{k}$ to describe the characteristics of each supplier $A_{i}$ under attributes $C_{j} (j = 1,2,3,4,5)$, and all decision-makers $E_{k}$ give their corresponding interval-valued intuitionistic fuzzy matrix $\tilde{R}^{k} = (\tilde{r}_{ij}^{k} )_{m \times n}$. The objective evaluation information obtained through statistical investigation is shown in Tables 1, 2, 3 and 4^42^, and the optimal supplier is determined.

**Table 1:** Interval-valued intuitionistic fuzzy matrix given by decision-maker $E_{1}$.

	*C*_1_	*C*_2_	*C*_3_	*C*_4_	*C*_5_
*A*_1_	([0.70,0.80],[0.10,0.15])	([0.60,0.75],[0.10,0.15])	([0.60,0.65],[0.15,0.20])	([0.70,0.80],[0.15,0.15])	([0.35,0.45],[0.35,0.50])
*A*_2_	([0.70,0.75],[0.15,0.20])	([0.65,0.80],[0.15,0.20])	([0.65,0.70],[0.25,0.30])	([0.50,0.55],[0.30,0.35])	([0.58,0.68],[0.22,0.32])
*A*_3_	([0.80,0.80],[0.10,0.15])	([0.70,0.80],[0.10,0.15])	([0.70,0.80],[0.10,0.15])	([0.65,0.70],[0.25,0.30])	([0.48,0.58],[0.29,0.39])
*A*_4_	([0.60,0.65],[0.25,0.30])	([0.70,0.85],[0.10,0.15])	([0.70,0.75],[0.15,0.20])	([0.50,0.55],[0.40,0.45])	([0.53,0.63],[0.16,0.22])

**Table 2:** Interval-valued intuitionistic fuzzy matrix given by decision-maker $E_{2}$.

	*C*_1_	*C*_2_	*C*_3_	*C*_4_	*C*_5_
*A*_1_	([0.70,0.75],[0.15,0.20])	([0.80,0.85],[0.15,0.20])	([0.70,0.75],[0.15,0.20])	([0.70,0.75],[0.20,0.25])	([0.45,0.53],[0.24,0.34])
*A*_2_	([0.75,0.75],[0.15,0.20])	([0.70,0.75],[0.10,0.20])	([0.65,0.75],[0.10,0.15])	([0.80,0.85],[0.10,0.20])	([0.35,0.45],[0.34,0.42])
*A*_3_	([0.65,0.70],[0.15,0.20])	([0.85,0.90],[0.10,0.15])	([0.60,0.70],[0.25,0.25])	([0.75,0.75],[0.15,0.20])	([0.50,0.60],[0.25,0.34])
*A*_4_	([0.75,0.80],[0.15,0.20])	([0.75,0.80],[0.05,0.10])	([0.55,0.60],[0.35,0.40])	([0.75,0.80],[0.10,0.15])	([0.27,0.37],[0.34,0.47])

**Table 3:** Interval-valued intuitionistic fuzzy matrix given by decision-maker $E_{3}$.

	*C*_1_	*C*_2_	*C*_3_	*C*_4_	*C*_5_
*A*_1_	([0.65,0.70],[0.20,0.25])	([0.80,0.85],[0.10,0.15])	([0.80,0.85],[0.10,0.15])	([0.80,0.85],[0.10,0.15])	([0.12,0.22],[0.58,0.66])
*A*_2_	([0.80,0.85],[0.10,0.12])	([0.75,0.85],[0.10,0.15])	([0.75,0.80],[0.15,0.20])	([0.70,0.80],[0.25,0.30])	([0.46,0.56],[0.18,0.29])
*A*_3_	([0.80,0.85],[0.10,0.15])	([0.65,0.65],[0.30,0.35])	([0.70,0.85],[0.10,0.15])	([0.62,0.65],[0.28,0.30])	([0.20,0.30],[0.40,0.50])
*A*_4_	([0.85,0.85],[0.10,0.15])	([0.60,0.75],[0.25,0.30])	([0.73,0.85],[0.12,0.17])	([0.70,0.70],[0.20,0.25])	([0.63,0.78],[0.05,0.12])

**Table 4:** Interval-valued intuitionistic fuzzy matrix given by decision-maker $E_{4}$.

	*C*_1_	*C*_2_	*C*_3_	*C*_4_	*C*_5_
*A*_1_	([0.60,0.65],[0.25,0.30])	([0.65,0.70],[0.25,0.30])	([0.65,0.65],[0.25,0.30])	([0.63,0.65],[0.24,0.30])	([0.30,0.40],[0.42,0.53])
*A*_2_	([0.70,0.75],[0.20,0.25])	([0.63,0.65],[0.27,0.30])	([0.58,0.60],[0.20,0.25])	([0.69,0.73],[0.17,0.25])	([0.56,0.66],[0.18,0.29])
*A*_3_	([0.59,0.65],[0.21,0.27])	([0.62,0.65],[0.22,0.35])	([0.60,0.75],[0.23,0.30])	([0.60,0.65],[0.25,0.30])	([0.12,0.22],[0.53,0.63])
*A*_4_	([0.85,0.85],[0.10,0.15])	([0.65,0.65],[0.28,0.35])	([0.67,0.70],[0.25,0.28])	([0.73,0.75],[0.21,0.25])	([0.46,0.56],[0.27,0.37])

**Step 1:** Calculate the decision-maker weight $\omega_{j}^{k}$ for each attribute $C_{j}$ of decision-makers $E_{k}$. For example, the decision-maker weight $\omega_{1}^{1}$ for each attribute $C_{j}$ of decision-maker $E_{1}$.

1. Calculate the similarity of decision-maker $E_{1}$ under attribute $C_{1}$

According to formula (6), the ideal solution vector $\tilde{r}_{1}$ under attribute $C_{1}$ is:

$$\begin{gathered} \tilde{r}_{1} = (\tilde{r}_{11} ,\tilde{r}_{21} ,\tilde{r}_{31} ,\tilde{r}_{41} ) = (([0.6625,0.7250],[0.1750,0.2250]),([0.7375,0.7750],[0.1500,0.1925]), \hfill \\ ([0.7100,0.7500],[0.1400,0.1925]),([0.7500,0.7875],[0.1500,0.2000])). \hfill \\ \end{gathered}$$

According to formulas (7)–(9), the individual negative ideal solution vector $\tilde{r}_{1}^{c}$, the individual left negative ideal solution vector $\tilde{r}_{1}^{L - }$ and the individual right negative ideal solution vector $\tilde{r}_{1}^{U - }$ under attribute $C_{1}$ are calculated as follows:

The individual negative ideal solution vector $\tilde{r}_{1}^{c}$ is

$$\begin{gathered} \tilde{r}_{1}^{c} = ([0.1750,0.2250],[0.6625,0.7250]),([0.1500,0.1925],[0.7375,0.7750]), \hfill \\ ([0.1400,0.1925],[0.7100,0.7500]),([0.1500,0.2000],[0.7500,0.7875]). \hfill \\ \end{gathered}$$

The individual left negative ideal solution vector $\tilde{r}_{1}^{L - }$ is

$$\begin{gathered} \tilde{r}_{1}^{L - } = ([0.6000,0.6500],[0.2500,0.3000]),([0.7000,0.7500],[0.2000,0.2500]), \hfill \\ ([0.5900,0.6500],[0.2100,0.2700]),([0.6000,0.6500],[0.2500,0.3000]). \hfill \\ \end{gathered}$$

The individual right negative ideal solution vector $\tilde{r}_{1}^{U - }$ is

$$\begin{gathered} \tilde{r}_{1}^{U - } = ([0.7000,0.8000],[0.1000,0.1500]),([0.8000,0.8500],[0.1000,0.1200]), \hfill \\ ([0.8000,0.8500],[0.1000,0.1500]),([0.8500,0.8500],[0.1000,0.1500]). \hfill \\ \end{gathered}$$

Then, the distance between the decision matrix of the decision-maker $E_{1}$ and each ideal solution vector is obtained by using formula (4):

$$d(\tilde{r}_{1}^{1} ,\tilde{r}_{1} ) = 0.0733,\,d(\tilde{r}_{1}^{1} ,\tilde{r}_{1}^{c} ) = 0.5555,\,d(\tilde{r}_{1}^{1} ,\tilde{r}_{1}^{L - } ) = 0.0882,\,d(\tilde{r}_{1}^{1} ,\tilde{r}_{1}^{U - } ) = 0.0811.$$

The similarity between the decision matrix of the decision-maker $E_{1}$ and the group decision matrix composed of all the decision-makers under the attribute $C_{1}$ is calculated by using the formula (10) $s_{1}^{1} = 0.9082$.

2. Calculate the proximity of decision-maker $E_{1}$ under attribute $C_{1}$

Combined with formula (11), the proximity between the attribute values of each scheme of decision-maker $E_{1}$ and decision-maker $E_{2}$ is:

$$\xi_{11}^{21} = 0.9500,\,\xi_{21}^{21} = 0.9646,\,\xi_{31}^{21} = 0.8882,\,\xi_{41}^{21} = 0.8677.$$

According to formula (12), the average proximity between vectors $\tilde{r}_{1}^{2}$ and $\tilde{r}_{1}^{1}$ is:

$$\eta (\tilde{r}_{1}^{2} ,\tilde{r}_{1}^{1} ) = \frac{1}{4}\sum\limits_{i = 1}^{4} {\xi_{i1}^{21} } = 0.9176.$$

Similarly,

$$\eta (\tilde{r}_{1}^{3} ,\tilde{r}_{1}^{1} ) = \frac{1}{4}\sum\limits_{i = 1}^{4} {\xi_{i1}^{31} } = 0.8955,\,\eta (\tilde{r}_{1}^{4} ,\tilde{r}_{1}^{1} ) = \frac{1}{4}\sum\limits_{i = 1}^{4} {\xi_{i1}^{41} } = 0.8668.$$

Using formula (13), the proximity between the decision maker $E_{1}$ and the other three decision makers on attribute $C_{1}$ is:

$$\eta_{1}^{1} = \frac{1}{3}\sum\limits_{l = 2}^{4} {\eta (\tilde{r}_{1}^{l} ,\tilde{r}_{1}^{1} )} = 0.8933.$$

3. Determine the weight of decision maker $E_{1}$ under attribute $C_{1}$

In formula (14), take $\lambda = 0.5$ and solve the combination weight $\tilde{\omega }_{1}^{1}$ of decision maker $E_{1}$ under attribute $C_{1}$.

$$\tilde{\omega }_{1}^{1} = 0.9007.$$

The combination weights of the other three decision-makers under the attribute $C_{1}$ can be obtained by using the similar calculation process. The calculation results are shown in Table 5.

**Table 5:** Similarity, proximity and combination weight of decision-makers under attribute $C_{1}$.

	*E*_1_	*E*_2_	*E*_3_	*E*_4_
Similarity	0.9082	0.9643	0.9254	0.9095
Proximity	0.8933	0.9235	0.9069	0.9027
Combination weight	0.9007	0.9439	0.9161	0.9061

Standardize the combination weights $\tilde{\omega }_{1}^{1}$,$\tilde{\omega }_{1}^{2}$,$\tilde{\omega }_{1}^{3}$,$\tilde{\omega }_{1}^{4}$ to get the decision-maker weights of all decision-makers under attribute $C_{1}$.

$$\omega_{1}^{1} = 0.2456,\,\omega_{1}^{2} = 0.2574,\,\omega_{1}^{3} = 0.2498,\,\omega_{1}^{4} = 0.2471.$$

In the same way, the decision-maker weights of all decision-makers on all attributes are calculated, as shown in Table 6.

**Table 6:** Decision makers' weights on all attributes.

	*C*_1_	*C*_2_	*C*_3_	*C*_4_	*C*_5_
*E*_1_	0.2456	0.2528	0.2521	0.2407	0.2612
*E*_2_	0.2574	0.2506	0.2492	0.2485	0.2314
*E*_3_	0.2498	0.2519	0.2479	0.2554	0.2480
*E*_4_	0.2471	0.2448	0.2507	0.2554	0.2595

**Step 2:** Using the n-dimensional IVIFWM operator in combination with the determined weight of the decision-maker, the individual decision matrix of the decision-maker is aggregated according to formula (19) to get a group decision matrix $\tilde{R} = (\tilde{r}_{ij} )_{m \times n}$, as shown in Table 7.

**Table 7:** Interval-valued intuitionistic fuzzy group decision matrix.

	*C*_1_	*C*_2_	*C*_3_	*C*_4_	*C*_5_
*A*_1_	([0.663, 0.725], [0.175, 0.225])	([0.713, 0.788], [0.149, 0.199])	([0.687, 0.724], [0.163, 0.213])	([0.708, 0.762], [0.173, 0.213])	([0.303, 0.399], [0.400, 0.510])
*A*_2_	([0.738, 0.775], [0.150, 0.192])	([0.683, 0.763], [0.154, 0.212])	([0.657, 0.712], [0.175, 0.225])	([0.674, 0.734], [0.204, 0.274])	([0.492, 0.592], [0.227, 0.328])
*A*_3_	([0.709, 0.750], [0.140, 0.193])	([0.705, 0.751], [0.180, 0.249])	([0.650, 0.775], [0.170, 0.213])	([0.654, 0.687], [0.233, 0.275])	([0.322, 0.422], [0.370, 0.468])
*A*_4_	([0.750, 0.788], [0.150, 0.200])	([0.675, 0.763], [0.169, 0.224])	([0.662, 0.725], [0.217, 0.262])	([0.672, 0.702], [0.226, 0.273])	([0.477, 0.589], [0.203, 0.292])

**Step 3:** According to formula (17), the weight $w_{j}$ of each attribute is calculated as follows:

$$w_{1} = 0.241,\,w_{2} = 0.236,\,w_{3} = 0.220,\,w_{4} = 0.215,\,w_{5} = 0.087.$$

The relative weight $w^{\prime}_{j}$ of attributes calculated by formula (18) and Step 4 is:

$$w^{\prime}_{1} = 1.000,\,w^{\prime}_{2} = 0.979,\,w^{\prime}_{3} = 0.914,\,w^{\prime}_{4} = 0.891,\,w^{\prime}_{5} = 0.361.$$

**Step 4:** According to the dominance matrix under attribute $C_{j}$ condition and referring to the value range of risk aversion coefficient, $\theta = (0.3,0.8,1.0,2.5,3.0,4.0)$.

For the attribute $C_{1}$, when $\theta = 1$, the dominance matrix $\varphi_{1} (A_{i} ,A_{k} )$ of the scheme $A_{i}$ relative to the scheme $A_{k}$ under the attribute $C_{1}$ is calculated by the formula (20) according to the $D_{HH}$ distance measure formula.

$$\varphi_{1} (A_{i} ,A_{k} ) = \left[ {\begin{array}{*{20}c} {0.0000} & { - 0.5577} & { - 0.4399} & { - 0.6029} \\ {0.1346} & {0.0000} & {0.0827} & { - 0.2323} \\ {0.1061} & { - 0.3429} & {0.0000} & { - 0.4122} \\ {0.1454} & {0.0560} & {0.0995} & {0.0000} \\ \end{array} } \right].$$

In the same way, the dominance matrices of the other attributes ($C_{2}$,$C_{3}$,$C_{4}$,$C_{5}$) can be obtained, which is not described in detail in the operation process due to limited space.

**Step 5:** According to formula (21), the overall superiority of the scheme $A_{i}$ relative to the scheme $A_{k}$ is obtained.

$$\varphi (A_{i} ,A_{k} ) = \left[ {\begin{array}{*{20}c} {0.0000} & { - 1.7679} & { - 1.3805} & { - 1.8685} \\ { - 0.9930} & {0.0000} & { - 0.1339} & { - 0.6346} \\ { - 0.7794} & { - 2.4909} & {0.0000} & { - 2.3727} \\ { - 1.1449} & {0.9689} & { - 0.1072} & {0.0000} \\ \end{array} } \right].$$

**Step 6:** According to formula (22), the comprehensive evaluation value of each scheme is obtained, and the schemes are sorted.

It is concluded from Table 8 that when the risk aversion coefficient $\theta$ takes different values, the ranking of the schemes is $A_{2} \succ A_{4} \succ A_{1} \succ A_{3}$. According to the ranking of the alternative schemes under different psychological indicators or risk preference degrees, and in combination with the stability presented in the decision-making process, the stable optimal scheme can be determined as $A_{2}$.

**Table 8:** Comparison of dominance of alternatives under different $\theta$ by two distance measure formulas.

*θ*	Distance measure	Calculation results of dominance of each scheme				Scheme ranking
		*A*_1_	*A*_2_	*A*_3_	*A*_4_	
0.3	*D*_*HH*_	0.1081	1	0	0.8993	*A*_2_ > *A*_4_ > *A*_1_ > *A*_3_
	*D*_*HE*_	0	1	0.1236	0.9418	*A*_2_ > *A*_4_ > *A*_3_ > *A*_1_
0.8	*D*_*HH*_	0.1469	1	0	0.8864	*A*_2_ > *A*_4_ > *A*_1_ > *A*_3_
	*D*_*HE*_	0	1	0.0974	0.9361	*A*_2_ > *A*_4_ > *A*_3_ > *A*_1_
1.0	*D*_*HH*_	0.1613	1	0	0.8816	*A*_2_ > *A*_4_ > *A*_1_ > *A*_3_
	*D*_*HE*_	0	1	0.0872	0.9338	*A*_2_ > *A*_4_ > *A*_3_ > *A*_1_
2.5	*D*_*HH*_	0.2524	1	0	0.8513	*A*_2_ > *A*_4_ > *A*_1_ > *A*_3_
	*D*_*HE*_	0	1	0.0145	0.9179	*A*_2_ > *A*_4_ > *A*_3_ > *A*_1_
3.0	*D*_*HH*_	0.2775	1	0	0.8429	*A*_2_ > *A*_4_ > *A*_1_ > *A*_3_
	*D*_*HE*_	0.0081	1	0	0.9136	*A*_2_ > *A*_4_ > *A*_1_ > *A*_3_
4.0	*D*_*HH*_	0.3216	1	0	0.8282	*A*_2_ > *A*_4_ > *A*_1_ > *A*_3_
	*D*_*HE*_	0.0490	1	0	0.9082	*A*_2_ > *A*_4_ > *A*_1_ > *A*_3_

As the difference of decision makers' risk preference in practical decision-making problems is objective and cannot be ignored, it is very reasonable and necessary to introduce risk aversion coefficient $\theta$ to quantify the degree of risk preference of decision makers in this study. On the premise of the determined risk aversion coefficient $\theta$, selecting any interval-valued intuitionistic fuzzy distance measure formula based on Hausdorff measure in the calculation does not affect the ranking of the alternatives, the selection of the distance measure formula has weak correlation with the determination of the optimal scheme, and the decision result has certain robustness.

If the $D_{HE}$ distance measure algorithm is adopted, the ranking of alternatives will also change obviously with the increase of the $\theta$ value, that is, the change of decision makers' attitude towards risks, the risk attitude of decision makers directly affects the whole decision-making behavior. When $\theta$ value is small, alternative $A_{3}$ is better than $A_{1}$; With the increase of $\theta$, alternative $A_{1}$ is superior to $A_{3}$. In the actual selection decision, if the supplier has unstable economic conditions and institutional sustainability^43^ and weak risk resistance, a smaller $\theta$ value can be selected in the calculation using this model. On the contrary, a larger parameter $\theta$ can be selected. This study shows that the emergency decision-making results under different risk attitudes can be obtained by changing the risk aversion coefficient $\theta$ to make the model more applicable. The risk attitude of decision makers is taken into account when sorting the schemes, and different decision options are provided for decision makers with different risk attitudes to better meet people's decision-making needs and enhance the flexibility of decision-making methods. Under the premise of maximizing the profits of supply chain enterprises, the research content herein provides ideas for formulating more accurate cooperation contracts to achieve win–win cooperation among supply chain members. As the research is based on the complexity of human decision-making behavior and risk attitudes, the drawn conclusions will be closer to the reality.

The actual application of a construction machinery manufacturing company in the central and southern China region shows that the optimal supplier well matches the actual supplier selection results. It is an enterprise with a good operation state in the construction industry and a long-term and stable cooperative relationship has been established with the machinery manufacturing company. This shows that this model is feasible to apply in actual supplier selection, which can provide reference for decision makers in supplier selection.

Thus it is clear that the method proposed in this study the related research results^42,44,45^ has the following advantages over:

1. The method proposed in this paper is compared with the group decision-making method based on simulation and IVIFS proposed in reference^42^, and the ranking result obtained by the combination of simulation and IVIFS is $A_{2} \succ A_{3} \succ A_{4} \succ A_{1}$. It is obvious that the optimal scheme obtained by the method proposed in this study is the same as that obtained by the group decision-making method in reference^42^, that is,$A_{2}$, but the order of each scheme is still different, mainly because the risk preferences of different decision-makers are taken into account in the TODIM method used in this paper, the influence of hesitation on decision-making results is quantified from the perspective of decision-makers, so the decision-making result is more in line with the actual decision-making situation.
2. It is more reasonable and practical to use the method proposed in this paper to determine the weight of decision-makers than the extended TOPSIS method used in reference^44^, because the weights of decision-makers determined by the extended TOPSIS method are the same for different attributes, and those by the method proposed in this paper are different for different attributes. In actual decision-making, since different attributes may involve different fields, and each decision maker is obviously only good at some fields but not all fields, it is more reasonable that decision makers have different weights under different attributes. In addition, the existing research results use similarity or proximity to determine the weight of decision makers, but the similarity is not necessarily high under great proximity. Therefore, this paper comprehensively uses similarity and proximity in the process of determining the weight of decision makers to better make full use of decision information, so that the determined decision maker weights are closer to the actual weights.
3. Compared with the extended TOPSIS method in literature^44^, the method proposed in this paper has strong distinguishability, which can help us determine the unique optimal solution for different risk preferences of decision makers. Nevertheless, the extended TOPSIS method may result in close comprehensive evaluation value of each scheme, which weakens the distinguishability of this method and makes it difficult to distinguish the schemes.
4. Compared with the method in reference^45^, the focus points of the two methods are obviously different. The method proposed by the former constructs the interval number fuzzy preference relation based on the unknown function and the fuzzy preference relation, and is applied to the MADM problem with only one decision maker, while the method proposed in this paper is applicable to the MAGDM problem with IVIFN whose attribute weight information is completely unknown. In addition, in the former method, the decision maker only needs to evaluate the preference relationship between schemes after pairwise comparison, while in the present method, each scheme is evaluated with respect to each attribute.

The paper improves the determination method based on decision maker's weight, and determines the decision maker's weight based on the fuzzy degree and uncertainty degree of the judgment information provided by the decision maker, which largely avoids subjectivity and authoritative monopoly. At the same time, considering the influence of different decision makers' risk preference degrees on the decision-making results, to avoid excessive weighting in an interval-valued intuitionistic fuzzy matrix, a measurement coefficient is introduced for the decision makers' risk preference degree. Decision makers can choose the corresponding decision-making results according to their own risk preferences, which can add more choices for decision-makers and increase the flexibility of the decision-making process. To sum up, the method proposed herein can not only provide the ranking results of decision makers in different risk preference styles, but also provide comprehensive decision-making results. This method has the advantages of objective and comprehensive consideration of problems and strong operability.

## Conclusions

With the rise of outsourcing and procurement globalization, supplier selection has become the focus of the current enterprise supply chain management because high-quality suppliers can effectively reduce the procurement cost of enterprises, gradually increase the flexibility of supply chain management and continuously improve the core competitiveness of enterprises. Under the environment of global procurement, outsourcing of non-core competence, internet and e-commerce, the decision-making of supplier selection is becoming more and more important and complex, and has always been the attention of the theoretical circle and the actual management workers. In this study, by considering the similarity and proximity at the same time, i.e. considering the similarity between the individual decision matrix and the group decision matrix of the decision maker, and referring to the proximity between the decision matrix of a single decision maker and the comprehensive decision matrix composed of other decision makers, a new method is proposed to determine the decision maker's weight for each attribute, which is more objective, comprehensive and persuasive in the discussion of the problem. Finally, an example of mechanical equipment supplier selection decision-making shows the feasibility and effectiveness of the selection evaluation criteria and MAGDM method, which can provide reference for decision-makers to select suppliers, and also provide an effective scientific method to solve the problem of intuitionistic fuzzy MAGDM. In the practical application process, it is necessary to establish relevant databases sand accumulate more experience. Appropriate model parameters can be selected to improve the performance of the group decision-making model of the IVIFS and TODIM method. The method proposed herein can more objectively and reasonably solve the interval-valued intuitionistic fuzzy MAGDM problem when the attribute weight and the decision maker's weight information are completely unknown. It can meet the needs of decision makers with different risk preferences, enrich and develop fuzzy group decision-making theory and method, broaden the application scope of group decision theory, thus providing decision makers with more practical and feasible decision-making reference.

## Data Availability

The data used in this paper are from the raw data of references^42,44^, this paper uses public data (unclassified data) for example analysis, in order to verify the feasibility and effectiveness of the proposed model and method.
